# Impact of early antiretroviral therapy, early life immunity and immune sex differences on HIV disease and posttreatment control in children

**DOI:** 10.1097/COH.0000000000000807

**Published:** 2023-07-06

**Authors:** Nicholas G. Herbert, Philip J.R. Goulder

**Affiliations:** Peter Medawar Building for Pathogen Research, Department of Paediatrics, University of Oxford, Oxford, United Kingdom

**Keywords:** CD8+ T-cells, early ART initiation, HLA class I, immune sex differences, natural killer cells, paediatric HIV

## Abstract

To review recent insights into the factors affecting HIV disease progression in children living with HIV, contrasting outcomes: following early ART initiation with those in natural, antiretroviral therapy (ART)-naive infection; in children versus adults; and in female individuals versus male individuals.

**Recent findings:**

Early life immune polarization and several factors associated with mother-to-child transmission of HIV result in an ineffective HIV-specific CD8+ T-cell response and rapid disease progression in most children living with HIV. However, the same factors result in low immune activation and antiviral efficacy mediated mainly through natural killer cell responses in children and are central features of posttreatment control. By contrast, rapid activation of the immune system and generation of a broad HIV-specific CD8+ T-cell response in adults, especially in the context of ‘protective’ HLA class I molecules, are associated with superior disease outcomes in ART-naive infection but not with posttreatment control. The higher levels of immune activation in female individuals versus male individuals from intrauterine life onwards increase HIV infection susceptibility in females *in utero* and may favour ART-naive disease outcomes rather than posttreatment control.

**Summary:**

Early-life immunity and factors associated with mother-to-child transmission typically result in rapid HIV disease progression in ART-naive infection but favour posttreatment control in children following early ART initiation.

## INTRODUCTION

The difference between early life and adult immune polarization depends fundamentally on the cytokines produced by innate immune cells in response to pathogen exposure. The Th1-supporting cytokines IFN-gamma and IL-12 facilitate the strong HIV-specific T-cell responses seen in adults that play a major part in immune control of HIV, whereas in children, these particular responses are not well supported and are consequently relatively weak [1–6]. However, the early life immune system is designed to serve other functions, such as a more regulated and tolerogenic immune environment with a relatively high level of regulatory T-cell activity, and also to promote other specific responses such as antibody production via Tfh-supporting cytokines, IL-6 and IL-21 [7]. Thus, the early life immune system is not ‘immature’ or systematically ‘weaker’ than the adult immune system but is designed differently to achieve optimal outcomes for the unique challenges faced in early life.

Indeed, in certain instances, compared with adults, early life immunity can generate superior immune responses, such as high-frequency and highly potent broadly neutralizing antibodies against HIV [8,9], and antibody responses to non-HIV and HIV vaccines [10–12]. The early life immune response can also achieve superior disease outcomes. A recent example is severe acute respiratory syndrome coronavirus 2 infection, where mortality in children aged 7 years, for example, was 25-fold lower than in an adults aged 30 years [13]. Chickenpox is a second example, where mortality in adults at least 20 years old is 21-fold higher than in children under 14 years of age [14]. By contrast, in HIV-1 infection, compared with adults, the early life immune system is less well adapted in the absence of antiretroviral therapy (ART) to achieve successful disease outcomes. However, in this review, we argue that, following early ART initiation, posttreatment control may be more easily attained in children than in adults. The reason for this is that the immune responses that most readily achieve immune control of HIV and minimize disease in natural, ART-naive infection – rapid activation of the immune system, and generation of a broad HIV-specific CD8+ T-cell response – are, in broad terms, the opposite of what is successful in achieving posttreatment control.

It is important to note that paediatric and adult HIV infection differ in one key respect in addition to the stage of immune ontogeny at which each becomes infected: the child is infected by the mother, whereas the adult is infected by an unrelated donor. This means that the virus that is transmitted from mother to child may be preadapted to the child's immune system as a result, for example, of HLA I-associated cytotoxic T-lymphocyte (CTL) escape in the mother involving HLA alleles shared with the child [15,16]. In addition, sex differences in the innate immune response typically result in stronger type I interferon (IFN-I) responses and superior disease outcomes among female individuals [17]. Thus, female infants living with HIV (LWH) are more likely to carry IFN-I-resistant viruses that have escaped the stronger innate immune system in the mothers making them more vulnerable to infection *in utero*[18]. As discussed below, this may have implications in children LWH, both for HIV pathogenesis in natural infection in the absence of ART, and for HIV cure potential following early ART initiation. 

**Box 1:**
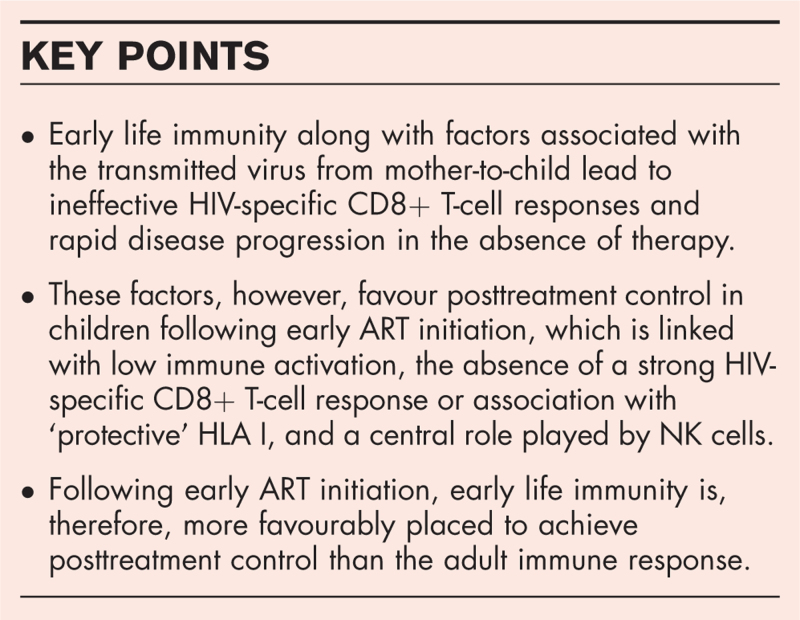
no caption available

## CD8+ T CELLS AND HIV DISEASE IN ANTIRETROVIRAL THERAPY-NAIVE INFECTION IN CHILDREN VERSUS ADULTS

The natural course of paediatric HIV infection is characterized by faster disease progression to AIDS and shorter time to death compared with adults. More than 50% of ART-naive children LWH have died by 2 years of age [19] whereas in ART-naive adults, the median survival time is 11 years [20]. In adult infection, slow disease progression and immune control of HIV are associated with polyfunctional, broad HIV-specific CD8+ T-cell responses [21–24], especially those targeting the highly conserved and abundant Gag capsid protein. The HLA-I molecules that are strongly linked with disease protection are, in Caucasian populations, HLA-B∗27 and HLA-B∗57 [25,26], and, in African populations, HLA-B∗57, HLA-B∗58:01 and HLA-B∗81:01 [27,28]. In each case, these HLA-I molecules present immunodominant CTL epitopes that are located within the capsid protein. Although escape mutations to evade these responses can be selected by the virus, in each case, they come at a considerable cost to viral replicative capacity [29–35]. Thus, immune control mediated by these HLA-I molecules results either from successful containment of viral replication via CTL-mediated killing, or via significant reduction in viral replicative capacity after the selection of immune escape.

In paediatric infection, as explained above, the HIV-specific T-cell response is not strongly supported by early life immune polarization. The low-frequency, narrowly based HIV-specific CD8+ T cells in the first 2 years of life do little to contain viral replication and it is during this time period that the majority of children LWH succumb to AIDS. In contrast to the protective part played by HLA-I molecules such as HLA-B∗27/57/58:01/81:01 in slowing disease progression in adults LWH, in children these HLA-I have little impact [36,37^▪^]. In part, this is because the virus transmitted from mother to child may have escape mutants that abrogate the beneficial effects of CTL responses restricted by these protective alleles, but principally it is because the HIV-specific CD8+ T-cell responses are so weak in early life.

## NK CELLS AND HIV DISEASE IN ANTIRETROVIRAL THERAPY-NAIVE INFECTION IN CHILDREN VERSUS ADULTS

NK cell activity in adult infection can protect against HIV disease progression, as shown by immunogenetic studies demonstrating that the combination of *Bw4-80I* alleles such as *HLA-B∗57* with high-expressing KIR3DL1 allotypes enhance the effects of HLA-B∗57 alone in slowing disease progression and improving control of viral load [38]. In addition, functional studies have demonstrated the ability of NK cells to exert immune control on HIV sufficient to drive the selection of escape variants capable of evading these antiviral NK responses [39–42]. However, these studies also indicate that, in adults, the dominant impact of protective HLA-I such as HLA-B∗27/57 is independent of these NK-cell interactions. By contrast, in ART-naive paediatric infection, NK responses have a greater impact on disease progression than HIV-specific CD8+ T-cell responses [37^▪^]. As stated above, HIV-specific CD8+ T-cell responses in the first few years of life are relatively weak and their impact on immune control becomes greater as children transition into adolescence. Consistent with this is the observation that HLA-B∗58:02, which is disease-susceptible in adult infection, and is Bw4-expressing and, therefore, serves as a ligand for KIR3DL1, does not carry any disease-susceptible effect in children until more than 10 years of age; by contrast, the other two principal disease-susceptible HLA-I in African populations, HLA-B∗18 and HLA-B∗45, which are both Bw6 expressing, have a substantial detrimental impact in the first 10 years of life as well [37^▪^].

## IMPACT OF IMMUNE SEX DIFFERENCES ON HIV INFECTION SUSCEPTIBILITY AND DISEASE

The very wide impact of immune sex differences on outcome from infections and immunizations, cancers, inflammatory conditions, and autoimmune disorders is becoming increasingly evident [17]. However, with respect to HIV, the very significant sex differences in outcomes have taken a long time to appreciate because studies in resource-rich countries have focused mainly on male individuals and in resource-poor countries on female individuals. Thus, it was not until 2017 that it became apparent that, among adults, females achieve elite control five times more frequently than male individuals [43]. Among children, elite control is 10 times less common than in adults [44], principally for the reasons described above related to the weakness of the HIV-specific CD8+ T-cell responses in early life. However, among the few paediatric elite controllers described, females outnumber males by 9 : 1 [44]. In adult and paediatric infection, viral setpoints are lower in female individuals compared with male individuals, in adults by 0.5 log [45]. In children, the superior immune control of HIV among female individuals becomes apparent from approximately 2 years of age; below that age, male individuals have lower viral loads [46,47]. These age-specific and sex-specific differences in immune control of HIV relate to the fine balance that exists between the beneficial impact of early immune activation of the innate immune system, and subsequently of adaptive immunity [48], and the negative effects of increased immune activation, which fuels the fire of viral replication and, in chronic infection, causes immune dysfunction and accelerates HIV disease progression [49–52]. The higher level of immune activation in female individuals compared with male individuals [53–55] that is present from intrauterine existence onwards [18] is associated with increased female susceptibility to in-utero infection [18,56–61] and, as stated above, higher viral loads in female individuals in the first 2 years of life. Beyond this age, the benefits of a stronger adaptive immune response, and more specifically the anti-HIV CD8+ T-cell response, increasingly are brought to bear on the virus.

The explanation for these observations lies in the fact that the female immune response is typically more activated and responds to pathogen exposure more rapidly and robustly. At the heart of this is the higher production of type I interferons (IFN-I) by plasmacytoid dendritic cells in female individuals compared with male individuals [53,62–64]. In addition to the direct antiviral effects of interferon-stimulated gene products (ISGs), such as the HIV-1 restriction factors tetherin, SAMHD1, TRIM5a, APOBEC3, and MX2, which are all ISGs [65], IFN-I activates and accelerates antiviral NK cell activity and also the HIV-specific adaptive immune response. The counterpoint to the more activated and aggressive immune response observed in female individuals compared with male individuals is increased immunopathology, with more rapid CD4 decline in chronic HIV infection in female individuals than male individuals for a given HIV viral load [45,53] and more autoimmune diseases such as systemic lupus erythematosus [66].

## COMBINED ANTIRETROVIRAL THERAPY-NAIVE IMMUNE CONTROL VERSUS POSTTREATMENT CONTROL OF HIV

Studies of the mechanisms underlying posttreatment control (PTC) in adult infection have unexpectedly pointed to striking differences with those that underpin immune control in ART-naive infection. First, whereas protective HLA-I are strongly associated with the latter, the HLA-I associated with PTC are those that are disease-susceptible in ART-naive infection [67,68]. In Caucasian populations, this is HLA-B∗35 (especially subtypes HLA-B∗35:02 and B∗35:03) [69,70], and among African cohorts, these are HLA-B∗18, B∗45 and B∗58:02 [27,28]. These disease-susceptible HLA-I molecules typically present a small number of HIV-specific epitopes, especially of Gag-specific epitopes. In addition, they tend to bind with strong avidity to the leucocyte immunoglobulin like receptors LILRB1 and LILRB2, that has the effect of dampening down the initiation of virus-specific CD8+ T-cell responses [71]. Second, consistent with the HLA-I associations, HIV-specific CD8+ T-cell responses among PTC are unremarkable [67,72^▪▪^]. Third, low levels of immune activation are associated with PTC [67,72^▪▪^], whereas immune control of ART-naive infection correlates with the speed and extent of immune activation [48]. It is no surprise, therefore, that, while adult elite controllers may exhibit the high levels of immune activation needed to control HIV infection, they also suffer an increased risk of coronary atherosclerosis [73]. Furthermore, the HLA-I molecules associated with high immune activation and elite control of HIV-1 infection are in many cases also linked with autoimmune diseases [74–76], the best characterized example being certain HLA-B∗27 subtypes with ankylosing spondylitis [76].

The fourth feature of PTC is the central role played by NK responses. Although NK cell activity contributes to immune control in ART-naive infection in adults [38–42] and plays a major part in preventing disease progression among children LWH [37^▪^], NK responses appear to play a central role in PTC, both in adults [72^▪▪^,^77^–^80^] and in children. In particular, it has been proposed that PTC is associated with an HLA-I signature that combines disease-susceptible HLA molecules with HLA haplotypes that mediate a KIR-biased education of NK cells [77]. The features contributing to such a haplotype are low-expressing HLA-A molecules, such as HLA-A∗03, A∗33, and A∗74 [81], HLA-B molecules expressing the Bw4 motif, HLA-B molecules expressing Threonine at residue-21 in the signal peptide, and HLA-C molecules within the C2 group. Low-expressing HLA-A molecules reduce the expression of HLA-E, which is the ligand for the NKG2A/CD94 heterodimer [82]. Nonamer peptides corresponding to residues -22 to -14 of the HLA-A, HLA-B, and HLA-C leader sequence bind HLA-E as long as the residue at HLA-21 is methionine, as is the case for all HLA-A and HLA-C molecules. The leader sequence in a portion of HLA-B molecules has threonine at residue -21 and this 9mer peptide does not bind to HLA-E [83]. Thus, threonine at residue -21 in the HLA-B leader sequence has the same effect as low HLA-A expression, by reducing the generation of the HLA-E-binding epitope from within the leader sequence, favouring KIR-education of NK cells. HLA-Bw4 is the ligand for KIR3DL1 and the C2 group of HLA-C molecules (which carry lysine at residue 80: HLA-C∗02, ∗04, ∗05, ∗06, ∗12, ∗16 : 02, ∗17, and ∗18) are ligands for the KIR2DL1 receptor. Thus, the HLA-I signature proposed to be associated with PTC is one that has a propensity to educate NK cells through KIR rather than NKG2A [84]. The fact that this HLA-I haplotype has been brought together in the course of evolution across many different populations [84] is strong evidence for its importance in the immune response. Interestingly, HLA-I haplotypes that educate NK cells through KIR are associated with reduced susceptibility to HIV infection in adults [85] as well as with more effective NK cell-mediated control of HIV both in adults [86] and in children LWH [37^▪^].

**FIGURE 1:**
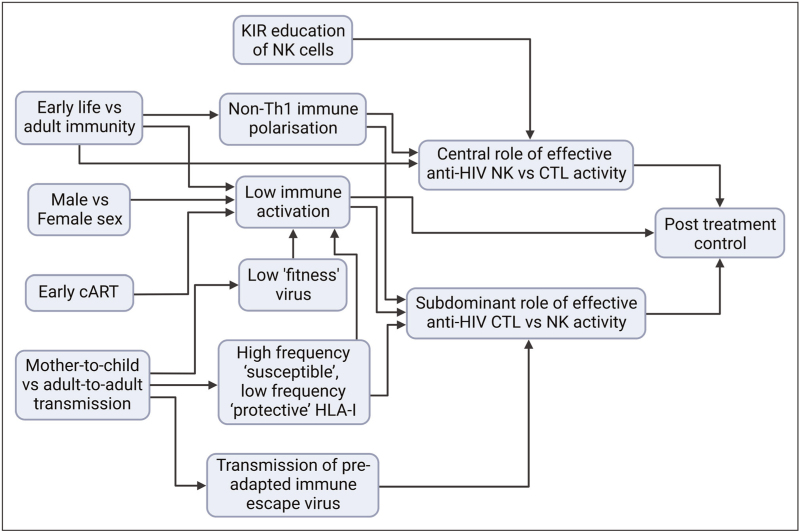
Key features predisposing to posttreatment control in children living with HIV.

Consideration of the differences between the factors that contribute to immune control of ART-naive and PTC control among adults highlights the fact that the early-life immune system is especially poorly designed to achieve ART-naive immune control of HIV (Fig. 1). However, it also prompts the hypothesis that the early-life immune system is especially well designed to achieve PTC. All four features of PTC identified above are supported by early-life immunity in children LWH. Indeed, children LWH are enriched for disease-susceptible HLA-I because mother-to-child transmission is strongly associated with high viraemia – and therefore, with disease-susceptible HLA-I in the mother [27]. As described above, the early-life immune polarization does not support virus-specific CD8+ T-cell responses but does promote a highly regulated, tolerogenic immune environment with low levels of immune activation. By contrast with CTL in early life, NK cell numbers are at their highest at birth, and NK responses are active and capable of effective HIV disease prevention from birth and potentially even *in utero*[37^▪^,^87^–^89^].

An additional, fifth, feature of PTC is early cART initiation. It has been shown both in nonhuman primate and human studies that early cART initiation, followed by an analytical treatment interruption (ATI), results in lower viral setpoints than arise in natural infection [90–92]. Early cART initiation suppresses the high levels of immune activation that rapidly drive immune dysfunction and also blocks the rapid selection of CTL escape viruses that occurs in natural infection, thus allowing a broad and effective antiviral immune response to develop during the initial period on cART. Following treatment interruption, the lower virus setpoint is in part because of virus-specific CD8+ T-cell activity, as demonstrated by CD8+ T-cell depletion studies in NHP [92] using the anti-CD8β monoclonal antibody, which does not deplete NK cells. However, as stated above, antiviral NK cell responses are a stronger correlate of posttreatment control than virus-specific CD8+ T-cell responses and, in early life, the dominant antiviral impact of NK cells may again place children LWH in a stronger position than adults to achieve functional cure following early cART initiation.

## VIRAL FACTORS IN PAEDIATRIC INFECTION

This review has focused on immune factors and the immunogenetic factors, especially HLA-I, KIR, and sex differences that slow HIV disease progression in children. An additional important factor is the impact of the virus transmitted to children compared with that transmitted to adults. The replicative capacity of the virus transmitted from mother-to-child is lower than that of the virus quasispecies circulating in the mother [93], whereas the replicative capacity of the virus transmitted from adult to adult is similar or somewhat higher in the recipient compared with that of the donor [94,95]. Transmission of a low replication virus is important because HIV disease progression is slower and immune activation and proviral DNA load are lower in the recipient [96]. In mother-to-child transmission, lower replicative capacity viruses are transmitted to female individuals [18], which may be related to the higher levels of immune activation observed in female individuals versus male individuals *in utero*, and also the selection of IFN-I-resistant viruses in mother-to-female foetal transmission. In the same way that CD8+ T-cell escape mutants often carry a cost to replicative capacity [29–35], innate immune escape variants may have similar effects on virus replication efficiency.

The consequence of lower ‘fitness’, IFN-I-resistant viruses being transmitted to female foetuses would, in ART-naive infection, likely result in superior outcomes among female children LWH. The higher frequencies of paediatric elite controllers who are female, the lower viral loads in female children above 2 years of age, and the female preponderance of paediatric nonprogressors [97] – healthy children with normal-for-age CD4 T-cell counts and low levels of immune activation despite persistently high viraemia [9] – are all consistent with this notion. However, the transmission of IFN-I-resistant viruses predominantly to females may undermine the ability of innate immunity to control viral rebound following treatment interruption, as the rebounding virus following ATI is highly IFN-I-resistant [98^▪^].

## CONCLUSION

Children LWH exhibit very different HIV disease outcomes compared with adults. In natural, ART-naive infection, early-life immunity and features linked with mother-to-child transmission mitigate against effective antiviral CD8+ T-cell responses, and precipitous disease progression is usually the outcome. By contrast, in adults, rapid CD8+ T-cell activation and suppression of viraemia as early as possible in acute infection is associated with much longer periods of disease-free infection. However, as observed in elite controllers, immune control may come at the cost of high immune activation, which itself brings well documented, significant disease in chronic infection.

The situation following early ART initiation is almost diametrically opposed to that in ART-naive infection. Recent studies have highlighted the key features associated with posttreatment control. Early ART initiation facilitates posttreatment control, but the salient immunological features that have been identified are low immune activation; the notable absence of an association with strong HIV-specific CD8+ T-cell activity or with ‘protective’ HLA-I; but a central role for antiviral NK responses. The immunogenetic link with posttreatment control is rather with ‘disease susceptible’ HLA-I, potentially through reduced immune activation via strong LILR-B2-binding affinities, and with HLA haplotypes that favour KIR education of NK cells and more effective antiviral NK responses in HIV infection. In these respects, early-life immunity appears better positioned than the adult immune response to achieve posttreatment control following early ART initiation. The immunotherapeutic interventions in addition to early ART initiation needed to achieve this are, suffice it to say, subject to very active current investigation.

## Acknowledgements

*This work is supported by the Wellcome Trust (PG WTIA Grant WT104748MA), the National Institutes of Health (PG RO1-AI133673, UM1-AI164566 and UO1-AI164566) and by a grant to P.G. through the EPIICAL Project (Early-treated Perinatally HIV-infected Individuals: Improving Children's Actual Life with Novel Immunotherapeutic Strategies). The EPIICAL Project is funded through an independent grant by ViiV Healthcare UK.*

### Financial support and sponsorship

*None.*

### Conflicts of interest

*There are no conflicts of interest.*

## REFERENCES AND RECOMMENDED READING

Papers of particular interest, published within the annual period of review, have been highlighted as:

▪ of special interest
▪▪ of outstanding interest
